# Grouping of Poorly Soluble Low (Cyto)Toxic Particles: Example with 15 Selected Nanoparticles and A549 Human Lung Cells

**DOI:** 10.3390/nano9050704

**Published:** 2019-05-06

**Authors:** Veno Kononenko, David B. Warheit, Damjana Drobne

**Affiliations:** 1 Department of Biology, Biotechnical Faculty, University of Ljubljana, Večna pot 111, 1000 Ljubljana, Slovenia; veno.kononenko@bf.uni-lj.si; 2 Warheit Scientific LLC, Wilmington, DE 19801, USA; david.warheit@gmail.com

**Keywords:** biopersistent particles, poorly soluble low toxicity particles (PSLTs), endo-lysosomal organelles, alveolar type II cells, fast screening tools

## Abstract

Poorly soluble, low (cyto)toxic particles (PSLTs) are often regarded as one group, but it is important that these particles can be further differentiated based on their bioactivity. Currently, there are no biological endpoint based groupings for inhaled nanoparticles (NPs) that would allow us to subgroup PSLTs based on their mode of action. The aim of this study was to group NPs based on their cytotoxicity and by using the in vitro response of the endo-lysosomal system as a biological endpoint. The endo-lysosomal system is a main cellular loading site for NPs. An impaired endo-lysosomal system in alveolar type II cells may have serious adverse effects on the maintenance of pulmonary surfactant homeostasis. The 15 different NPs were tested with human lung adenocarcinoma (A549) cells. The highly soluble NPs were most cytotoxic. With respect to PSLTs, only three NPs increased the cellular load of acid and phospholipid rich organelles indicating particle biopersistence. All the rest PSLTs could be regarded as low hazardous. The presented in vitro test system could serve as a fast screening tool to group particles according to their ability to interfere with lung surfactant metabolism. We discuss the applicability of the suggested test system for bringing together substances with similar modes-of-action on lung epithelium. In addition, we discuss this approach as a benchmark test for the comparative assessment of biopersistence of PSLTs.

## 1. Introduction

Safety testing of every unique particle for their potential adverse effects is virtually impossible. Therefore, innovative science-based approaches that support safety assessments are needed. When performing studies on nanoparticle (NP) hazard effects, we need to consider the relevance and reliability of using experimental methods as well as legislative and ethical aspects of animal studies [1]. In order to make a transition from the use of experimental animals to animal alternatives, we need to develop effective and relevant in vitro protocols to discriminate biologically more potent particle-types from those of low hazard [2]. Utilizing in vitro methods with human cells follows the principle of the 3Rs (replacement, refinement and reduction of laboratory animals) and has the potential to improve the relevance of nanosafety assessment for humans [1]. It has been shown, for example, that rats are an inappropriate model for predicting human lung cancer risk following chronic particle overload inhalation exposures [3]. In vitro studies are easier to control and reproduce, faster to perform, and more cost-effective than animal studies [4]. The in vitro studies are essential for the mechanistic understanding of NP-cell interactions [5]. 

The grouping of NPs aims to make the hazard identification and assessment of a variety of nanotechnology-enabled products more efficient by bringing together substances with similar hazardous profiles [6]. There are many concepts for the grouping of NPs, including intrinsic NP properties, specific types of NP use, exposure route, uptake, biodistribution, biopersistence, release, and cellular and organism-level toxic effects [7,8]. Poorly soluble, low (cyto)toxic particles (PSLTs) can be defined as poorly soluble particles of low (cyto)toxicity, which is equivalent to poorly soluble particles (PSPs). The ECETOC Task Force defines PSPs as particles that have dissolution half-lives measured in artificial fluids longer than macrophage mediated clearance times. According to this definition, macrophage clearance rather than particle dissolution determines the particle residence time in the lung. PSPs (and PSLTs) are also viewed as particles with no specific inherent toxicity [3,9]. PSLTs are often regarded as one group, but it is important that within this group, particles can be differentiated based on their variations in bioactivity [10]. Currently there are no biological endpoint based groupings for inhaled PSLTs based on their mode of action. 

The lung represents both, an important entry route and a barrier for unintentionally inhaled or deliberately administered NPs [11,12]. Thus, great efforts are currently being made to understand the interactions between NPs and lung cells [13]. Particles that enter the lung alveoli can be phagocytosed and removed from the lung alveolar epithelium by alveolar macrophages. Nevertheless, the macrophage clearance of inhaled particles is not efficient in all situations [14]. It was reported that the macrophage clearance of poorly soluble particles is impaired when the average composite phagocytized volume exceeds 6% of the normal macrophage volume (in the case of lung particle overload) [15]. In addition, some NPs used for medical applications are engineered to avoid macrophage clearance [16]. NPs which escape macrophage clearance (intentionally or unintentionally) and remain in the alveolar space for a longer period are likely to interact with the alveolar epithelium [17]. These particles are of particular interest in nanosafety assessments. 

Alveolar epithelium consists of squamous alveolar type I cells that comprise the major gas exchange surface, and of cuboidal alveolar type II cells that act as progenitors for alveolar type I cells and are responsible for the synthesis, storage and secretion of lung surfactant. Lung surfactant is a predominantly phospholipid substance that is stored in alveolar type II cells in special cell organelles-lamellar bodies, which are exocytosed into the alveolar lumen and are reorganized to form a surfactant film in the air-liquid interface. The surfactant film enables normal breathing and prevents lung collapse by reducing surface tension of the alveolar hypophase. Lung surfactant also plays an important role in the pulmonary immune defense [18]. A substantial proportion of lung surfactant in the hypophase is recycled through reuptake by alveolar type II cells and this endocytic path is also involved in the internalization of NPs into alveolar type II cells [19,20,21]. Internalized NPs in alveolar type II cells were frequently observed inside the endo-lysosomal system that has an essential role in the phospholipid (lung surfactant) metabolism [22,23]. It was reported that A549 human lung cancer cells, which are frequently used as an in vitro model for alveolar type II cells, internalize NPs and keep them inside early endosomes, multivesicular bodies, lysosomes, and lamellar bodies [24,25,26]. Internalized NPs can influence the cellular load of lamellar bodies, which can have functional consequences [19,27,28,29]. The interference of NPs with the endo-lysosomal system in model lung epithelium cells can be used as a key event responsible for an adverse outcome due to NP exposure and thus the basis for classification and grouping of NPs according to their tissue specific mode of action (interference with lipid/surfactant metabolism and accumulation of biopersistent particles).

In the study presented here, we used human lung adenocarcinoma cells (A549 cells) that are among the most frequently used lung epithelial models and share several properties with alveolar type II cells [30,31,32]. In addition, A549 cells have retained the ability to increase the phospholipid surfactant pool in response to harmful substances [33]. We compared the potential of 15 different NPs to interfere with the endo-lysosomal system, lipid surfactant metabolism and cytotoxicity. Cell staining with neutral red dye was used to observe an increased cellular load of acid endo-lysosomal organelles indicating intracellular accumulation of NPs. Staining with the LipidTOX^TM^ Green phospholipidosis detection reagent was used to evaluate cellular phospholipid accumulation (phospholipid rich organelles like multivesicular bodies, lamellar bodies and autophagic vacuoles). Cytotoxicity was evaluated by the MTT assay. In our previous work, we confirmed a correlation between the NP interference with lipid surfactant metabolism and the intracellular accumulation of NPs that caused an increased cellular load of acid endo-lysosomal and phospholipid rich organelles [19]. In the study presented here we selected a broad variety of NPs (see Table 1), which vary in the surface chemistry or core composition, some are known to have a high dissolution rate, while others are less able to dissolve in biological media. The aim of this approach was to group NPs with different physicochemical characteristics by their tissue specific biological effects (interference with the endo-lysosomal system and accumulation of biopersistent particles). We discuss the use of interference of NPs with the endo-lysosomal system (affected lipid surfactant metabolism, intracellular accumulation of NPs) as a key event leading to an adverse outcome and thus a basis for bringing together substances with a similar mode-of-action on lung epithelium. In addition, we discuss this suggested approach as a benchmark testing protocol for the comparative assessment of biopersistence of PSLTs.

## 2. Materials and Methods

### 2.1. Chemicals

The HCS LipidTOX^TM^ Green phospholipidosis detection reagent was from Life Technologies (Carlsbad, CA, USA). Cell culture media and all other chemicals used in our experiments were from Sigma-Aldrich (Steinheim, Germany), unless stated otherwise. 

### 2.2. Preparation and Characterization of Nanoparticle Suspensions

In our study, we used 15 different NPs (Table 1). We used different silicon dioxide NPs (pure SiO_2_, amino-functionalized SiO_2_-NH_2_ and carboxylic acid-functionalized SiO_2_-COOH) that we received from the Joint Research Centre in Italy (cooperation in the European project NanoMILE). We used different superparamagnetic iron oxide NPs. Superparamagnetic maghemite NPs were a courtesy of our research partners from the Jozef Stefan Institute (dr. Slavko Kralj). We used uncoated maghemite NPs (γ-Fe_2_O_3_), maghemite NPs coated with silica (γ-Fe_2_O_3_+SiO_2_) and γ-Fe_2_O_3_+SiO_2_ NPs with additionally modified surface with amino or carboxyl groups (γ-Fe_2_O_3_+SiO_2_-NH_2_, γ-Fe_2_O_3_+SiO_2_-COOH). In addition to maghemite NPs, we used different superparamagnetic iron oxide NPs with mixed maghemite/magnetite structure (Mix γ-Fe_2_O_3_/Fe_3_O_4_ and Mix γ-Fe_2_O_3_/Fe_3_O_4_, which were stabilized with citrate or malate) that we received from a Friedrich-Alexander University Erlangen-Nürnberg (dr. Stefanie Klein). In addition to the above mentioned SiO_2_ and iron oxide NPs, we also used Buckminsterfullerenes (C_60_), copper NPs (Cu), copper oxide NPs (CuO), zinc oxide NPs (ZnO nano) and zinc oxide microparticles (ZnO micro) that were purchased from Sigma-Aldrich (Steinheim, Germany).

Stock suspensions of NPs at 10 mg·mL^−1^ were prepared in deionized water (MilliQ, Millipore, Billerica, MA, USA [pH = 5.7, ρ = 18.5 MΩ·cm]). Before the experiments, the stock suspensions of NPs were mixed and sonicated in an ultrasonic water bath (15 min, 250 W, 50 Hz, Sonis 2GT, Iskra Pio, Slovenia). Sonicated suspensions of NPs were used to prepare working NPs suspensions in the cell culture medium. Primary sizes of NPs in suspensions were characterized using the transmission electron microscopy (TEM; JEOL 2100, Tokyo, Japan). The hydrodynamic diameter of the NPs in their aqueous suspensions (50 µg·mL^−1^) was obtained using the dynamic light scattering (DLS; Analysette 12 DynaSizer, Fritsch GmbH, Idar-Oberstein, Germany). The 50 µg·mL^−1^ NP suspensions were monitored with electro-kinetic measurements of the zeta-potential (ZetaPALS potential analyzer, Brookhaven Instruments Corp, Holtsville, NY, USA).

### 2.3. Cell Culture

A549 cells were cultured in Dulbecco’s modified Eagle’s medium (DMEM), supplemented with 4 mM L-glutamine and 5% (v/v) fetal bovine serum (FBS). Cells were grown at 37 °C in a humidified atmosphere with 5% CO_2_. A549 cells were confirmed to be Mycoplasma negative using the MycoAlert^TM^ Kit (Lonza, Basel, Switzerland), following the manufacturer’s protocol.

### 2.4. MTT Assay

The cytotoxicity of selected NPs was evaluated by the MTT assay, which is used for the evaluation of mitochondrial dehydrogenase activities in living cells. It reflects the number of viable, metabolically active cells. The MTT assay was performed as described previously [19]. Briefly, A549 cells (2.2 × 10^4^ cells·cm^−2^) were seeded in transparent 96-well plates. After a 24-h incubation, the cells were treated with NPs (1–50 µg·mL^−1^). After a 24-h exposure, the 0.5 mg mL^−1^ MTT reagent was added and after a 3-h incubation, the formed formazan crystals were diluted with dimethyl sulfoxide (DMSO). The absorbance of the reduced MTT was measured spectrophotometrically by a microplate reader (BioTek, Cytation 3, Bad Friedrichshall, Germany) at 570 nm. All cytotoxicity experiments were performed at least twice in five replica wells. In order to test the potential NP interference with the MTT assay, we included different controls as suggested by Drasler et al. (2017) [34]. To test if NPs can interfere with optical reading, we measured 570 nm absorbance of cell samples with NPs without the MTT reagent. Measured values were subtracted from the absorbance of samples with cells. Catalytic properties of NPs, which could result in the reduction of the MTT reagent, were assessed by measuring samples with NPs, reagent, and without cells. None of the used NPs caused the MTT reduction. In addition, results of the MTT assay were verified by the resazurin assay (protocol described in Kononenko and Drobne, 2019 [35]). 

### 2.5. The Estimation of Phospholipid Rich Organelle Quantity

Staining with the HCS LipidTOX^TM^ Green phospholipidosis detection reagent (Life Technologies, Carlsbad, CA) is used to evaluate the cellular phospholipid accumulation (phospholipid rich organelles like multivesicular bodies, lamellar bodies and autophagic vacuoles). A549 cells (4.5 × 10^4^ cells·cm^−2^) were seeded in 24-well plates with inserted sterile coverslips. After a 24-h incubation, allowing the cells to adhere, the cells were exposed to all selected NPs (Table 1) for 48 h. In the preliminary testing, we applied all NPs at a concentration 50 µg·mL^−1^. However, some particles (Cu, CuO, ZnO nano, ZnO micro, Mix γ-Fe_2_O_3_/Fe_3_O_4_, Mix γ-Fe_2_O_3_/Fe_3_O_4_-citrate, Mix γ-Fe_2_O_3_/Fe_3_O_4_-malate) proved to be cytotoxic at this concentration, making the evaluation of LipidTOX staining unreliable. For these particles, we performed tests with an NP concentration at 10 µg·mL^−1^. As a positive control (increase in phospholipid rich organelle quantity) we used 30 µM propranolol. The cells were exposed to the LipidTOX detection reagent, according to the manufacturer’s recommendations, at the same time the NP treatment started. After a 48-h exposure, cells attached to the coverslips were washed with Dulbecco’s Phosphate-Buffered Saline (DPBS) and fixed with 3.5% formaldehyde for 30 min. After fixation, coverslips were rinsed with DPBS and put on the microscope slide with a drop of Vectashield antifade mounting medium with DAPI (Vector laboratories, Burlingame, USA). Prepared microscope slides were observed with an epifluorescence microscope (Axio Imager.Z1; Carl Zeiss, Jena, Germany). From each slide, fluorescence of at least 100 cells was evaluated, according to the evaluation scale elaborated previously [19]. For each treatment condition, four independent biological repetitions were performed. Results are presented as an average evaluation score.

### 2.6. The Estimation of the Cellular Load of Acid Organelles

Staining with neutral red dye is used to detect acid cellular compartments in viable cells. In order to evaluate if the cellular load of acid cell organelles changes after a 48-h incubation of A549 cells with selected NPs, we stained exposed cells with neutral red dye that becomes trapped inside acid cellular compartments. A549 cells (4.5 × 10^4^ cells·cm^−2^) were seeded in 24-well plates with inserted sterile coverslips. After a 24-h incubation, the cells were exposed to 15 different NPs (Table 1) for 48 h. In the preliminary testing, all NPs at a concentration 50 µg·mL^−1^ were used. However, some particles (Cu, CuO, ZnO nano, ZnO micro, Mix γ-Fe_2_O_3_/Fe_3_O_4_, Mix γ-Fe_2_O_3_/Fe_3_O_4_-citrate, Mix γ-Fe_2_O_3_/Fe_3_O_4_-malate) proved to be highly cytotoxic at this concentration. For these particles, we repeated testing with the concentration 10 µg·mL^−1^. As a positive control (anticipated increase of cellular acid organelle load) we used 30 µM propranolol. After treatment with NPs, cells were exposed to neutral red dye (0.04 mg·mL^−1^) for 3 h. The cells on coverslips were washed with DPBS and visually observed with the differential interference contrast (DIC) microscopy (Axio Imager.Z1; Carl Zeiss, Jena, Germany).

### 2.7. Statistical Analysis

The data from the LipidTOX staining and cytotoxicity experiments was expressed as the arithmetic mean ± standard deviation (SD) and was statistically analyzed by a one-way ANOVA with Bonferroni’s post hoc test for multiple comparisons. A *p* value lower than 0.05 was considered statistically significant. All statistical analyses were performed using Microsoft Excel 2013 (Microsoft Corporation, USA) and the GraphPad Prism software (GraphPad Software, San Diego, CA, USA). 

## 3. Results

### 3.1. Particle Characteristics

As revealed by the TEM analysis, the size distribution for both ZnO nano and ZnO micro was broad, with the average diameter of 72 ± 46 nm and 237 ± 119 nm, respectively. the size distribution of C_60_, Cu and CuO NPs was also broad, with the average diameter of 26 ± 20 nm, 105 ± 52 nm and 130 ± 80 nm. In contrast, the size distribution for all iron oxide NPs and for all SiO_2_ NPs was quite narrow. The average diameter for γ-Fe_2_O_3_ was 12 ± 2 nm, for γ-Fe_2_O_3_+SiO_2_ was 19 ± 2 nm, for γ-Fe_2_O_3_+SiO_2_-COOH was 28 ± 3 nm, for γ-Fe_2_O_3_+SiO_2_-NH_2_ was 30 ± 3 nm, for Mix γ-Fe_2_O_3_/Fe_3_O_4_ was 14 ± 3 nm, for Mix γ-Fe_2_O_3_/Fe_3_O_4_-citrate was 12 ± 3 nm, and for Mix γ-Fe_2_O_3_/Fe_3_O_4_-malate was 11 ± 2 nm. Results of DLS and zeta potential measurements are presented in Table 1. 

### 3.2. Cytotoxicity of Tested Particles

**Figure 1:**
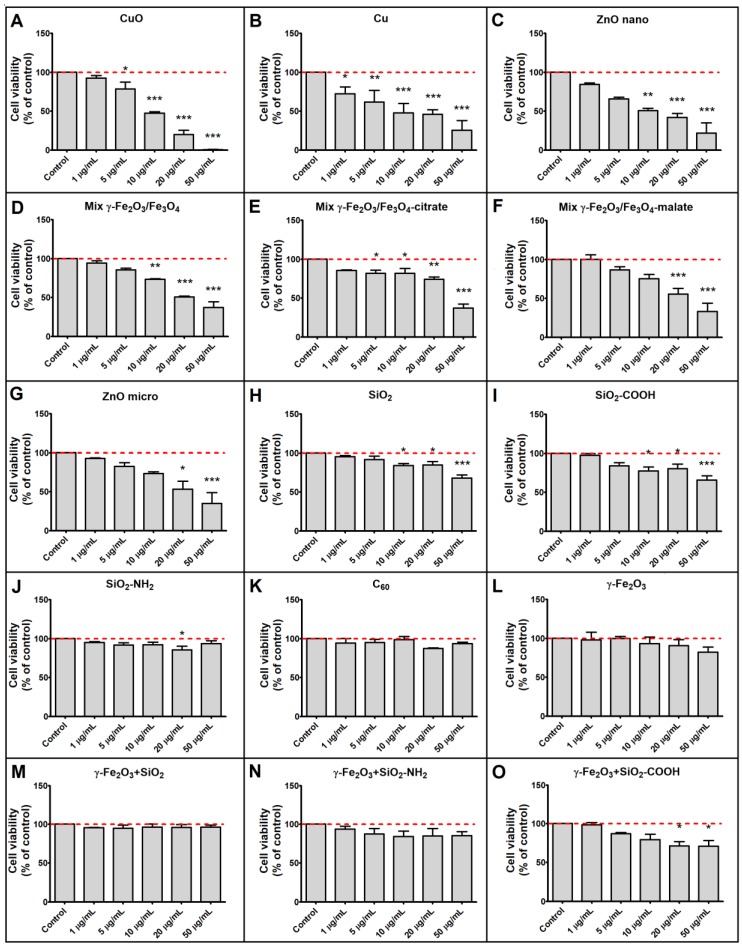
Cytotoxicity of nanoparticles (NPs). Cytotoxicity of (**A**) CuO, (**B**) Cu, (**C**) ZnO nano, (**D**) Mix γ-Fe_2_O_3_/Fe_3_O_4_, (**E**) Mix γ-Fe_2_O_3_/Fe_3_O_4_-citrate, (**F**) Mix γ-Fe_2_O_3_/Fe_3_O_4_-malate, (**G**) ZnO micro, (**H**) SiO_2_, (**I**) SiO_2_-COOH, (**J**) SiO_2_-NH_2_, (**K**) C_60_, (**L**) γ-Fe_2_O_3_, (**M**) γ-Fe_2_O_3_+SiO_2_, (**N**) γ-Fe_2_O_3_+SiO_2_-NH_2_, (**O**) γ-Fe_2_O_3_+SiO_2_-COOH. Cytotoxicity of NPs to A549 cells was evaluated by the MTT assay, after a 24-h exposure. Results are expressed as mean (+SD) percentage of untreated control cells in a representative experiment performed twice in five replica wells. Asterisk indicates a significant difference with respect to untreated control cells (dashed line; * equals *p* < 0.05; ** equals *p* < 0.01; *** equals *p* < 0.001; one-way ANOVA, Bonferroni’s post hoc test).

A549 cells were exposed to selected NPs for 24 h and their cytotoxic effect was assessed by the MTT assay. The viability of A549 cells reduced by more than 50% compared to control levels after exposure to 50 µg·mL^−1^ of CuO, Cu, ZnO nano, ZnO micro, Mix γ-Fe_2_O_3_/Fe_3_O_4_, Mix γ-Fe_2_O_3_/Fe_3_O_4_-citrate and Mix γ-Fe_2_O_3_/Fe_3_O_4_-malate. Exposure to 50 µg·mL^−1^ of γ-Fe_2_O_3_, γ-Fe_2_O_3_+SiO_2_, γ-Fe_2_O_3_+SiO_2_-COOH, γ-Fe_2_O_3_+SiO_2_-NH_2_, C_60_, SiO_2_, SiO_2_-NH_2_ and SiO_2_-COOH did not decrease cell viability below 50% of the control level. Cytotoxicity of selected NPs at different exposure concentrations are presented in Figure 1.

### 3.3. Cellular Load of Phospholipid Rich Organelles

**Figure 2:**
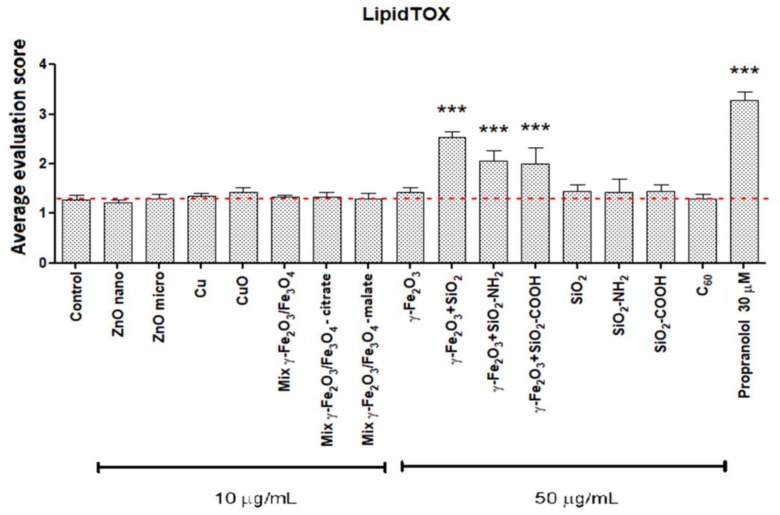
Cellular quantity of phospholipid rich organelles in A549 cells after a 48-h incubation with nanoparticles. The fluorescence intensity of the LipidTOX dye was microscopically estimated. For each treatment, four independent biological repeats, where at least 100 cells were evaluated, were performed. Results are presented as an average evaluation score (+SD). Asterisk indicates a significant difference with respect to untreated control cells (dashed line; *** equals *p* < 0.001; one-way ANOVA, Bonferroni’s post hoc test).

**Figure 3:**
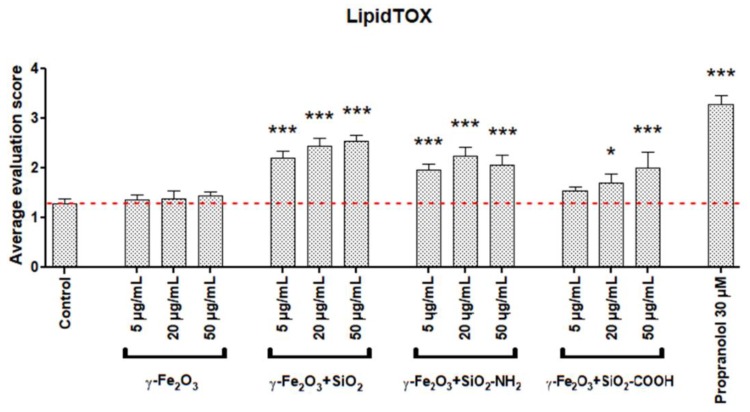
Concentration dependent increase in cellular quantity of phospholipid rich organelles in A549 cells after a 48-h incubation with maghemite nanoparticles. For each treatment condition, four independent biological repeats, where at least 100 cells were evaluated, were performed. Results are presented as an average evaluation score (+SD). Asterisk indicates a significant difference with respect to untreated control cells (dashed line; * equals *p* < 0.05; *** equals *p* < 0.001; one-way ANOVA, Bonferroni’s post hoc test).

**Figure 4:**
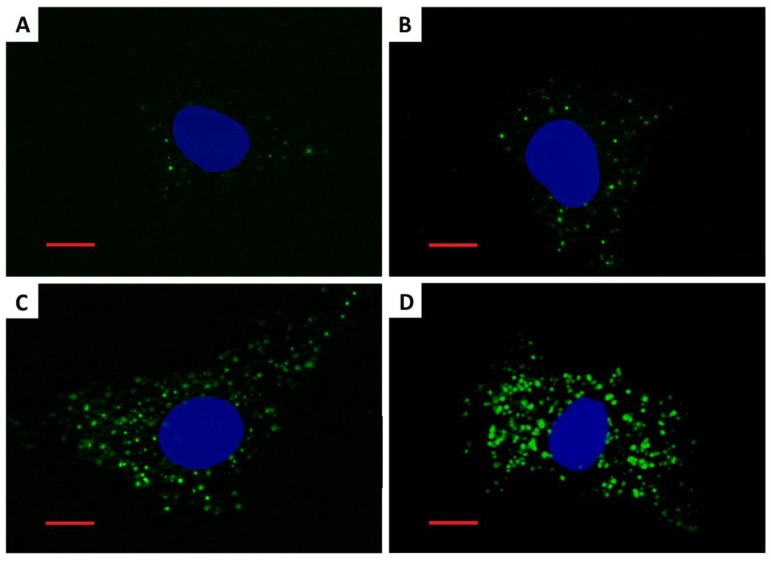
Fluorescence images of A549 cells stained by the LipidTOX dye after a 48-h incubation. (**A**) Untreated control cell; (**B**) cell treated with 20 µg·mL^−1^ γ-Fe_2_O_3_+SiO_2_-COOH; (**C**) cell treated with 20 µg·mL^−1^ γ-Fe_2_O_3_+SiO_2_-NH_2_; (**D**) cell treated with 20 µg·mL^−1^ γ-Fe_2_O_3_+SiO_2_. Green fluorescence represents phospholipid rich organelles. Blue fluorescence represents cell nuclei. Scale bar = 10 µm.

Staining with the LipidTOX detection reagent revealed an increased cellular quantity of phospholipid rich organelles in A549 cells only after treatment with silica coated maghemite NPs (γ-Fe_2_O_3_+SiO_2_, γ-Fe_2_O_3_+SiO_2_-COOH, γ-Fe_2_O_3_+SiO_2_-NH_2_; Figure 2, Figure 3, Figure 4 and Figure S1). We showed that the increased LipidTOX fluorescence was concentration dependent (Figure 3). All other NPs did not increase intracellular LipidTOX fluorescence (Figure 2), showing that no significant accumulation of phospholipid rich organelles after exposure to NPs occurred.

### 3.4. Cellular Load of Acid Organelles

**Figure 5:**
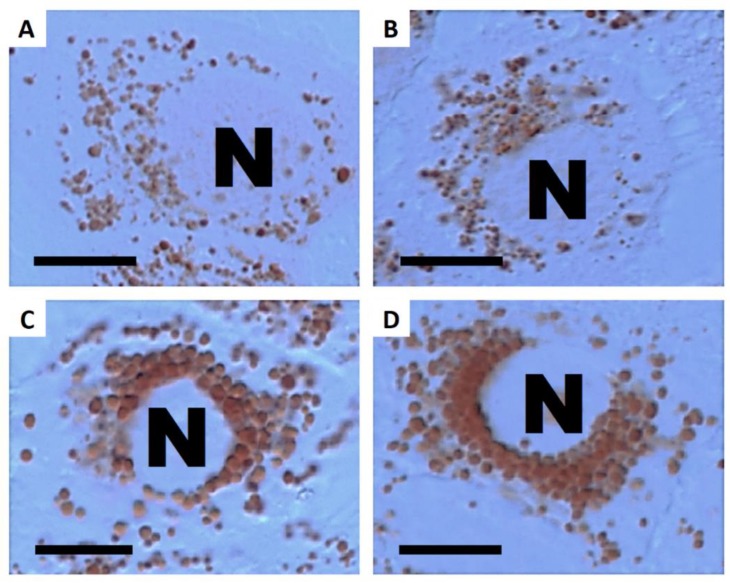
Differential interference contrast (DIC) images of A549 cells stained by neutral red dye after a 48-h incubation. (**A**) Untreated control cell; (**B**) cell treated with 20 µg·mL^−1^ γ-Fe_2_O_3_+SiO_2_-COOH; (**C**) cell treated with 20 µg·mL^−1^ γ-Fe_2_O_3_+SiO_2_-NH_2_; (**D**) cell treated with 20 µg·mL^−1^ γ-Fe_2_O_3_+SiO_2_. Unstained nuclei (*N*) are surrounded by acid organelles stained red. Scale bar = 10 µm.

Staining with neutral red dye has revealed an increased cellular load of acid organelles (late endosomes and lysosomes) in A549 cells that were treated only with silica-coated maghemite NPs (γ-Fe_2_O_3_+SiO_2_, γ-Fe_2_O_3_+SiO_2_-COOH, γ-Fe_2_O_3_+SiO_2_-NH_2_; Figure 5and Figure S2). No other NPs had an observable effect on the cellular load of acid organelles. These results showed that all NPs, which increased the cellular quantity of phospholipid rich organelles (Figure 2) also increased the cellular load of acid organelles. 

NPs that induced increased cellular numbers of phospholipid rich organelles or acid organelles were not highly cytotoxic (Table 2). 

## 4. Discussion

It is known that NP interactions with alveolar epithelial cells can induce oxidative stress and consequently inflammation, DNA damage, loss of cell membrane integrity, modulation of protein synthesis and organelle damage, all leading to a loss of cell viability [31]. In our study, from the 15 selected NPs (Table 1) the most cytotoxic were those that quickly dissolve in an aqueous environment (ZnO, Cu, CuO). Cytotoxicity could arise as a result of a high free ion concentration in cell culture medium or in endo-lysosomal organelles [36,37]. In addition, we observed a higher cytotoxicity to ZnO nano than to ZnO micro, showing that the size of chemically identical particles is an important factor determining cytotoxicity. There are two likely explanations for the observed NP size dependent effect. The first being that bigger NPs dissolve more slowly [38]. The second reason could be due to size dependent particle endocytosis [39,40]. Inside the cells, the acidic interior of the endo-lysosomal compartments accelerates ion dissolution, leading to a high local concentration of released ions [41,42]. These released ions can increase the permeability of the lysosomal membrane, causing an imbalance in ionic homeostasis, oxidative stress and cytotoxicity [43,44]. As expected, NPs that are known to be either slightly soluble (like iron oxide particles) or insoluble in aqueous media (like SiO_2_ and C_60_) were only moderately cytotoxic to A549 cells (Figure 1; Table 2). We detected moderate cytotoxicity to Mix γ-Fe_2_O_3_/Fe_3_O_4_, Mix γ-Fe_2_O_3_/Fe_3_O_4_-citrate, Mix γ-Fe_2_O_3_/Fe_3_O_4_-malate and SiO_2_ particles (Figure 1). Functionalization of SiO_2_ NPs with carboxylic group (SiO_2_-COOH) had only a minor and non-significant effect on cytotoxicity, while functionalization with amino group (SiO_2_-NH_2_) significantly reduced the cytotoxicity of silica NPs. Maghemite NPs (γ-Fe_2_O_3_) and different silica coated maghemite NPs (γ-Fe_2_O_3_+SiO_2_, γ-Fe_2_O_3_+SiO_2_-NH_2_, γ-Fe_2_O_3_+SiO_2_-COOH), did not reduce cell viability over 30% at concentration 50 µg·mL^−1^ (Table 2). We measured low, statistically insignificant cytotoxicity of C_60_ (Figure 1), consistent with the results of Wang et al. (2014), who found that C_60_ did not reduce the viability of A549 cells up to 200 µg·mL^−1^ [45]. All this favors the explanation that particle solubility is the main characteristic of particles which determines their cytotoxicity, consistent with the findings of Horie et al. (2012), who compared the physicochemical properties of 24 different NPs with their cellular effects in vitro on A549 cells [46]. Similar results on both A549 cells and macrophage-like THP-1 cells were reported also by Jeong et al. (2018), who showed that fast dissolving NPs do not always have similar toxic potentials compared to their constituent metal chlorides, and that this may be due to the differences in their intracellular uptake [47]. 

The effect of poorly soluble particles on A549 cells was not be as pronounced and the mode of action is less understood compared to highly soluble particles. Once endocytosed, slightly soluble or insoluble NPs could either accumulate in the acid endo-lysosomal organelles or leave the cell by exocytosis [39]. Accumulation of non-degraded material can lead to an increase in the size and number of acid endo-lysosomal organelles [48]. Our results on A549 cells showed that among all selected NPs (Table 1) only γ-Fe_2_O_3_+SiO_2_, γ-Fe_2_O_3_+SiO_2_-NH_2,_ and γ-Fe_2_O_3_+SiO_2_-COOH increased the cellular load of acid endo-lysosomal organelles (Figure 5 and Figure S2) and the same NPs increased the quantity of phospholipid rich organelles (Figure 2, Figure 3 and Figure 4). 

**Figure 6:**
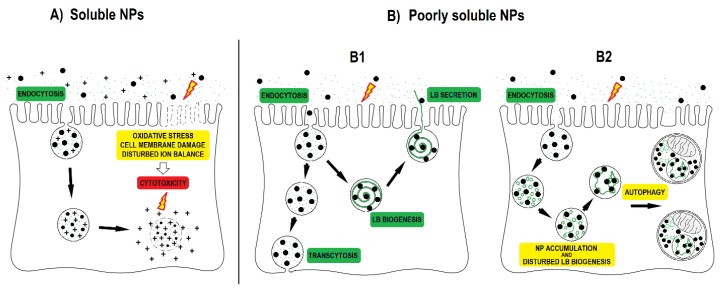
Nanoparticle (NP) interactions with alveolar type II cells. (**A**) Soluble NPs such as Cu, CuO and ZnO can release ions in the cell medium and even more in acid endo-lysosomal organelles after endocytosis due to the low pH. NPs and released ions in the cell medium as well as inside the cell can cause oxidative stress, damage cell membranes, and disturb the cellular ion balance, all of which can lead to cytotoxicity; (**B**) poorly soluble NPs are endocytosed by alveolar type II cells. Poorly soluble NPs can also induce oxidative stress and cytotoxicity, but usually require higher concentrations than more soluble NPs; (**B1**) internalized NPs can be secreted out of the cell together with lung surfactant packed in lamellar bodies or can be translocated across the cell by transcytosis; (**B2**) internalized NPs that enter the endo-lysosomal system can interfere with the synthesis and degradation of phospholipids, and with lamellar body biogenesis. NP interference with lamellar body biogenesis leads to autophagic degradation of defective lamellar bodies filled with NPs and to the accumulation of surfactant phospholipids in autophagic vacuoles (based on research of Kononenko et al., 2017 [19]).

NPs that accumulate in acid endo-lysosomal organelles may disturb the degradation of cellular or endocytosed phospholipids in the lysosome, leading to an intracellular accumulation of phospholipids. An increase in the synthesis of phospholipid rich lung surfactant can also lead to an increase in the phospholipid rich organelle content. An increased synthesis of lung surfactant in alveolar type II cells was suggested to be a protective mechanism to reduce the harmful effects of particles [49]. In addition to NPs impacts on phospholipid metabolism, the cause for the increased content of phospholipid rich organelles might be due to NP interference with lamellar body biogenesis. On the basis of TEM analyses in our previous study, we documented that endocytosed NPs by A549 cells disturb lamellar body biogenesis by interfering with the packing of surfactant into concentric membranes of lamellar bodies [19]. Defective lamellar bodies filled with NPs and with disorganized phospholipid membranes were sequestered in autophagic vacuoles, the number of which increased enormously. The increased amounts of cellular phospholipids correlated with the NPs exposure concentration. In the case of NPs where no increase in the cellular load of acid and phospholipid rich organelles was observed (Figure 2, Table 2), we speculate that endocytosis was balanced (proportional) with exocytosis of NPs via the secretion of lamellar bodies or via transcytosis (Figure 6) [17,20,39]. 

We detected increased LipidTOX fluorescence in A549 cells that were exposed to silica-coated maghemite NPs, but not in A549 cells that were exposed to SiO_2_, SiO_2_-NH_2_ and SiO_2_-COOH (Figure 2). This indicates that surface chemistry alone is not the main factor determining an NPs potential to accumulate in cells and to increase the cellular quantity of phospholipid rich organelles. We speculate that the potential of NPs to increase the cellular amount of acid endo-lysosomal and phospholipid rich organelles dependents on endocytosis of NPs and on further NP-cell processing (exocytosis or retention; Figure 6).

We organized the 15 tested NPs into four distinct groups according to their mode of action: (1) NPs that are highly cytotoxic and not biopersistent i.e., do not increase the load of endo-lysosomal organelles (Cu, CuO, ZnO); (2) NPs that are moderately cytotoxic and not biopersistent (Mix γ-Fe_2_O_3_/Fe_3_O_4_, SiO_2_, SiO_2_-COOH); (3) NPs that are not cytotoxic but are biopersistent and have the ability to interfere with surfactant metabolism (γ-Fe_2_O_3_+SiO_2_, γ-Fe_2_O_3_+SiO_2_-COOH, γ-Fe_2_O_3_+SiO_2_-NH_2_); and (4) NPs with no observed effects (C_60_, SiO_2_-NH_2_, γ-Fe_2_O_3_). The first group is comprised of soluble NPs. The poorly soluble NPs can be divided into the three remaining groups based on their potential to interfere with the endo-lysosomal system i.e., based on their biopersistence.

According to the principles of the European Chemicals Agency (ECHA), chemical substances that have similar properties may be placed in groups for the purpose of risk evaluation. Oomen et al. (2014, 2015) among others, stated that the term “group” or “category” represents a number of NPs which share commonalities relevant to their risk [8,50]. In line with this, Landvik et al. (2018) made a comprehensive summary of different available strategies for grouping NPs [51]. They have recognized five different commonalities of NPs, relevant for grouping:

- Fibrous versus non-fibrous or granular particles;
- Biopersistent versus non-biopersistent materials;
- Materials with high solubility versus low solubility;
- Chemically reactive versus chemically non-reactive materials;
- Materials with high toxicity versus low toxicity.

There is some variation in the combination of these characteristics between different grouping proposals but the basic principles are the same. The in vitro test system presented in our work could serve as a fast screening tool for grouping particles according to the most relevant characteristics of NPs, with an emphasis on biopersistence. It has been shown that in vitro systems utilizing A549 cells can provide meaningful nanotoxicity data [52]; but nevertheless, the reliability of our in vitro test approach should be confirmed with in vivo experiments. The proposed endpoints, based on alterations in the endo-lysosomal system supports the adverse outcome pathway (AOP) concept. The endo-lysosomal system is a known target and main cellular loading site for NPs. In the case of alveolar type II cells, an affected endo-lysosomal system could serve as a key event leading to a compromised lipid (surfactant) metabolism, causing adverse pulmonary effects.

This test system could also form an essential part of benchmark in vitro testing. Benchmark testing implies the comparative assessment of new materials against ‘benchmark materials’, which were previously tested and evaluated according to the selected criteria. Such benchmark testing could constitute an important pillar in the safety assessment of the abundance of NPs and their modifications.

## 5. Conclusions

Based upon our results on A549 human lung cells, NPs could be organized into four distinct groups based on their cytotoxicity profiles (i.e., as determined using the MTT assay) and biopersistence (shown as an increased cellular load of acid and phospholipid-rich organelles). The first group is made up of the highly soluble particles (Cu, CuO, ZnO) that were the most cytotoxic among the tested NPs. Poorly soluble NPs can be divided into three remaining groups: Moderately cytotoxic NPs (Mix γ-Fe_2_O_3_/Fe_3_O_4_, SiO_2_); low cytotoxic (C_60_, SiO_2_-NH_2_, SiO_2_-COOH, γ -Fe_2_O_3_); low cytotoxic, which are biopersistent and have a potential to interfere with surfactant metabolism (γ-Fe_2_O_3_+SiO_2_, γ-Fe_2_O_3_+SiO_2_-COOH, γ-Fe_2_O_3_+SiO_2_-NH_2_). The identified biological effects did not significantly correlate with any of the measured physicochemical characteristic of NPs (NP primary size, hydrodynamic diameter, zeta potential). This test system could form an essential part of the endpoint based grouping of NPs, as a benchmark in vitro test of new materials against ‘benchmark materials’ and in comparative safety assessment studies of existing NPs and their modifications.

## Figures and Tables

**Table 1:** Characteristics of nanoparticles. Average primary size was determined by the transmission electron microscopy (TEM). Average hydrodynamic diameter and zeta potential was determined in aqueous suspensions at 50 µg·mL^−1^.

Nanoparticles	Average Diameter of Nanoparticles [nm]	Average Hydrodynamic Diameter [nm]	Zeta Potential [mV]
ZnO nano	72	85	−13
ZnO micro	237	113 ^1^	−17
γ-Fe_2_O_3_	12	108	−16
γ-Fe_2_O_3_+SiO_2_	19	118	−42
γ-Fe_2_O_3_+SiO_2_-COOH	28	128	−35
γ-Fe_2_O_3_+SiO_2_-NH_2_	30	135	+10
Mix γ-Fe_2_O_3_/Fe_3_O_4_	14	110	−12
Mix γ-Fe_2_O_3_/Fe_3_O_4_-citrate	12	85	−25
Mix γ-Fe_2_O_3_/Fe_3_O_4_-malate	11	90	−22
C_60_	26	185	−36
Cu	105	NA ^2^	−17
CuO	130	NA ^2^	−24
SiO_2_	30	40	−31.5
SiO_2_-NH_2_	30	42	−20
SiO_2_-COOH	30	42	0

^1^ Before the measurement, we observed intensive sedimentation of the particles. Measurement did not include sedimented particles. ^2^ Measurement was not possible, due to the instability of particle suspension.

**Table 2:** Comparison of measured biological effects for selected nanoparticles (NPs). Cytotoxicity of NPs at 50 µg·mL^−1^ was measured by the MTT assay after a 24-h exposure of A549 cells. Cellular quantity of phospholipid rich organelles and cellular load of acid organelles were microscopically evaluated in LipidTOX or neutral red stained A549 cells after a 48-h treatment with 50 µg·mL^−1^ NPs. For particles that proved to be highly cytotoxic at 50 µg·mL^−1^ (Cu, CuO, ZnO nano, ZnO micro, Mix γ-Fe_2_O_3_/Fe_3_O_4_, Mix γ-Fe_2_O_3_/Fe_3_O_4_-citrate, Mix γ-Fe_2_O_3_/Fe_3_O_4_-malate), we performed phospholipid rich organelles and acid organelles evaluations at NP concentration 10 µg·mL^−1^.

Nanoparticles	Cytotoxicity over 70 %	Cytotoxicity over 50 %	Cytotoxicity over 30 %	Increased Phospholipid Rich Organelles	Increased Acid Organelles
Cu	+	+	+	−	−
CuO	+	+	+	−	−
ZnO nano	+	+	+	−	−
ZnO micro	−	+	+	−	−
Mix γ-Fe_2_O_3_/Fe_3_O_4_	−	+	+	−	−
Mix γ-Fe_2_O_3_/Fe_3_O_4_-citrate	−	+	+	−	−
Mix γ-Fe_2_O_3_/Fe_3_O_4_-malate	−	+	+	−	−
SiO_2_	−	−	+	−	−
SiO_2_-COOH	−	−	+	−	−
SiO_2_-NH_2_	−	−	−	−	−
C_60_	−	−	−	−	−
γ -Fe_2_O_3_	−	−	−	−	−
γ-Fe_2_O_3_+SiO_2_	−	−	−	+	+
γ-Fe_2_O_3_+SiO_2_-COOH	−	−	−	+	+
γ-Fe_2_O_3_+SiO_2_-NH_2_	−	−	−	+	+

## Supplementary Materials

The following are available online at https://www.mdpi.com/2079-4991/9/5/704/s1, Figure S1: Fluorescence images of A549 cells stained by the LipidTOX dye after a 48-h incubation; Figure S2: Phase contrast images of A549 cells stained by neutral red dye after a 48-h incubation.
