# Supplementary material for: Grouping of Poorly Soluble Low (Cyto)Toxic Particles: Example with 15 Selected Nanoparticles and A549 Human Lung Cells
Source: Nanomaterials (Basel). 2019 May 6;9(5):704. doi: 10.3390/nano9050704 (PMC6566622; doi:10.3390/nano9050704)
Supplement: Supplementary file 1 [file nanomaterials-09-00704-s001.pdf]

SUPPLEMENTARY MATERIAL

Grouping of poorly soluble low (cyto)toxic particles: example with 15 selected nanoparticles and A549 human lung cells

Veno Kononenko [1], David B. Warheit [2] and Damjana Drobne [1,*]

[1] Department of Biology, Biotechnical Faculty, University of Ljubljana, Večna pot 111, 1000 Ljubljana, Slovenia; veno.kononenko@bf.uni-lj.si

[2] Warheit Scientific LLC; david.warheit@gmail.com

[*] Correspondence: damjana.drobne@bf.uni-lj.si

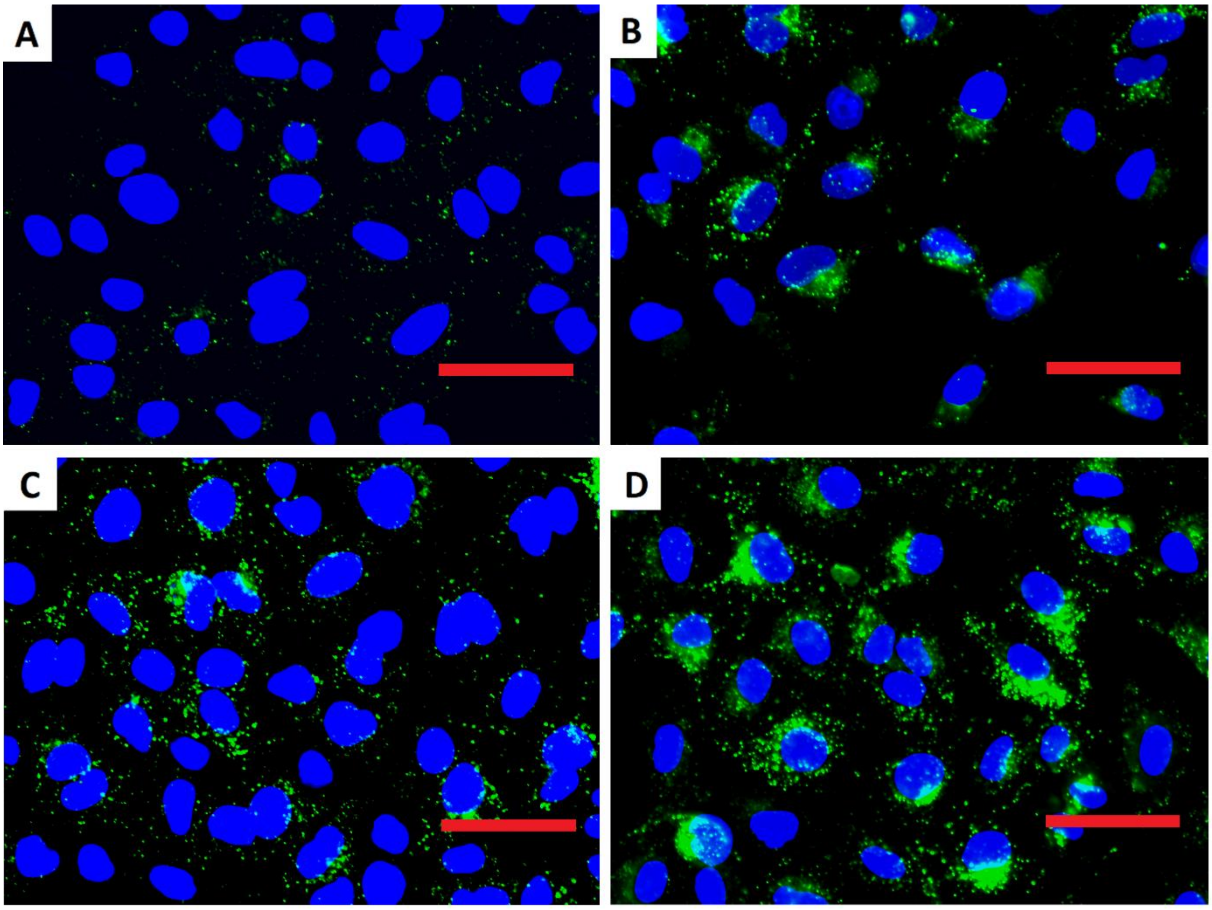

Figure S1. Fluorescence images of A549 cells stained by the LipidTOX dye after a 48-hour incubation. (**A**) Untreated control cells. (**B**) Cells treated with 20 µg mL^{-1} γ-Fe_2O_3+SiO_2-COOH. (**C**) Cells treated with 20 µg mL^{-1} γ-Fe_2O_3+SiO_2-NH_2. (**D**) Cells treated with 20 µg mL^{-1} γ-Fe_2O_3+SiO_2. Green fluorescence represents phospholipid rich organelles. Blue fluorescence represents cell nuclei. Scale bar = 50 µm.

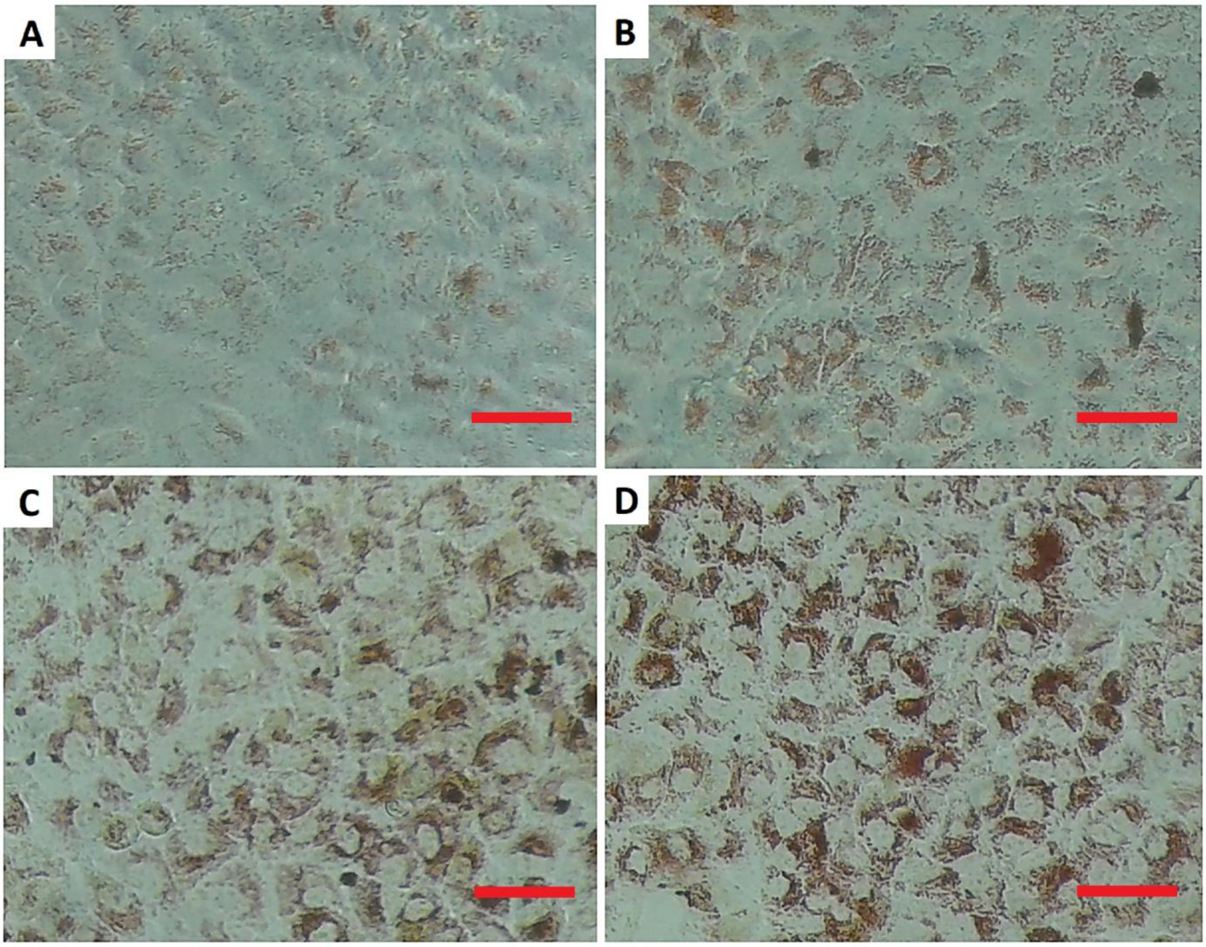

Figure S2. Phase contrast images of A549 cells stained by neutral red dye after a 48-hour incubation. (**A**) Untreated control cells. (**B**) Cells treated with 20 µg mL^{-1} γ-Fe_2O_3+SiO_2-COOH. (**C**) Cells treated with 20 µg mL^{-1} γ-Fe_2O_3+SiO_2-NH_2. (**D**) Cells treated with 20 µg mL^{-1} γ-Fe_2O_3+SiO_2. Unstained nuclei are surrounded by acid organelles stained red. Scale bar = 50 µm.
